# Supplementary material for: Stacking Model‐Based Classifiers for Dealing With Multiple Sets of Noisy Labels
Source: Biom J. 2025 Mar 12;67(2):e70042. doi: 10.1002/bimj.70042 (PMC11898607; doi:10.1002/bimj.70042)
Supplement: Supplementary file 1 — Supporting Information [file BIMJ-67-e70042-s001.zip › density-based-ensemble-model-main/competitors_real_data/netlab-master/netlab-master/help/mlpevfwd.htm]

Netlab Reference Manual mlpevfwd


# mlpevfwd

## Purpose

Forward propagation with evidence for MLP

## Synopsis

```
[y, extra] = mlpevfwd(net, x, t, x_test)
[y, extra, invhess] = mlpevfwd(net, x, t, x_test, invhess)
```

## Description

`y = mlpevfwd(net, x, t, x_test)` takes a network data structure
`net` together with the input `x` and target `t` training data
and input test data `x_test`.
It returns the normal forward propagation through the network `y`
together with a matrix `extra` which consists of error bars (variance)
for a regression problem or moderated outputs for a classification problem.
The optional argument (and return value)
`invhess` is the inverse of the network Hessian
computed on the training data inputs and targets. Passing it in avoids
recomputing it, which can be a significant saving for large training sets.

## See Also

`fevbayes`

---

**Pages:**
Index


---

Copyright (c) Ian T Nabney (1996-9)
